# Safety and Efficacy of Propranolol in Comparison With Combination of Fentanyl and Ketamine as Premedication in Cataract Surgery Under the Topical Anesthesia

**DOI:** 10.5539/gjhs.v7n6p88

**Published:** 2015-03-30

**Authors:** Farhad Fazel, Hamidhajigholam Saryazdi, Leila Rezaei, Mohammad Mahboubi

**Affiliations:** 1 Eye Research Center, Esfahan University of Medical Sciences, Esfahan, Iran; 2 Esfahan University of Medical Sciences, Esfahan, Iran; 3 Kermanshah University of Medical Sciences, Kermanshah, Iran; 4 Abadan School of Medical Sciences, Abadan, Iran; 5 Kermanshah University of Medical Sciences, Kermanshah, Iran

**Keywords:** propranolol, ketamine, fentanyl, cataract surgery, premedication

## Abstract

This study evaluated the safety and effects of propranolol as a premedication before cataract surgery and compared them with the usual combination doses of fentanyl and ketamine. Among all reffered patients to Feiz Hospital of Esfahan for cataract surgery, 122 patients between Mar to Sep 2010 were enrolled in this study and randomly allocated into one of the following equal groups: 40 mg propranolol, 2 hours before surgery and combination of 15 mg ketamine and 50 µg fentanyl l. 5 min before surgery. The ability to control of hemodynamic instabilities caused by stress and to gain patients satisfaction was compared between two groups. Also, the efficacy of each premedication to control of hemodynamic changes during surgery were evaluated and compared. No significant differences were seen in the patients satisfaction and controlling of stress induced hemodynamic changes between two groups (P>0.05). However, patients in ketamine + fentanyl group showed more nausea and less pain during and after surgery. Moreover, no significant adverse effects were reported during and after the surgery. Our results demonstrated that propranolol can be used safely as a premedication in cataract surgery in the comparable efficacy to ketamine plus fentanyl premedication.

## 1. Introduction

Cataract is a clouding of the lens which can lead to visual disability. There are several causes for cataract formation include age (the most common cause), trauma, radiation, genetic, systemic diseases, drugs and etc. Age-related cataracts are responsible for about 51% of world blindness (about 20 million people) in 2010 (Osei, 2011). Cataract extraction surgery is the most common ophthalmic surgery and commonly done under local anesthesia (Bakry, 2012). This is usually ’outpatient’ and performed using topical anesthesia and about 90% of patients can achieve a corrected vision of 20/40 or better after surgery (Bollinger, 2008).

The principle goal of sedation for cataract surgery is to prepare the patient to stay calm during retrobulbar injection and surgery (Khezri, Oladi, & Atlasbaf, 2013). Using of local anesthesia for ophthalmic operations provides clear immobile field with good patient and surgeon cooperation (Ayoglu, 2007). Therefore, most cataract surgeries in recent years are performed by phacoemulsification under topical anesthesia. Topical anesthesia protects patients from the possibility of globe perforations, optic nerve injury, and risk of respiratory arrest (Khezri & Merate, 2013). Moreover, general anesthesia with opioids provides good perioperative analgesia in ocular surgeries but is associated with the risk of respiratory depression and postoperative emesis (Sethi, 2013).

Propranolol (Inderal) is a sympatholytic nonselective β-blocker which used to treat of hypertension, anxiety, and panic disorder (Kornischka, 2007). It has been reported that premedication with oral propranolol 10 mg before hypotensive anesthesia with sodium nitroprusside is safe and effective to reduce reflex tachycardia and the amount of sodium nitroprusside used (Apipan & Rummasak, 2010). Usually propranolol is used by paediatric cardiologists in children as young few weeks to treat tachydysrhythmias, idiopathic hypertrophic subaortic stenosis, paroxysmal hypoxaemic spells, long Q-T syndrome, and congenital heart failure at doses up to 8mg/kg/day in divided doses (Reddy & Cornish, 2012). Totally, cataract surgery by using local anesthesia and premedication with oral sedative drugs such as ketamine and fentanyl is highly recommended. However, the number of reports about using propranolol in oral route as cataract surgery premedication is scarce.

Therefore, in this present study, due to inexpensiveness and also availability of propranolol in comparison to other premedication agents, we investigated the effects of propranolol on stress induced hemodynamic instabilities, patients satisfaction and hemodynamic changes during surgery in patients that underwent cataract surgery under topical anesthesia and comparing with ketamine plus fentanyl premedication.

## 2. Materials and Methods

In a randomized controlled trial (RCT) with simple random sample selection, with assuming that satisfaction probability was 50% (p=0.5) and α=0.05, and d=0.18, sample volume of 61 patients were calculated for each group. Our inclusion criteria were cooperation in clinical examination, first cataract surgery, having Persian language and filling conscious satisfaction form. Patients with body condition grades IV, V, and VI (based on ASA category), need to general anesthesia, myocardial infarction history (from 6 months ago), combined surgery procedure, history of bronchospasm, asthma and COPD, hypotension, cardiac block grade II and III, uncontrolled heart failure, concurrent use of sedative, hypnotic, opioid, beta blocker and ergotamine, and history of cerebrovascular accident (CVA) were excluded from our study.

Propranolol (Inderal) 40 mg tablet 2 hours before cataract surgery in group I and combination of 15 mg ketamine and 50 µg fentanyl, 5 min before cataract surgery in groups II were administered. All patients were underwent small incision phacoemulsification surgery by one surgeon in Feiz Medical Center. Local anesthesia was performed with 0.5% lidocaine drip. Also, 1% mydriacyl eye drops contain the active ingredient tropicamide was used for inducing mydriasis. Each patient was monitored during the surgery and excluded from the study if needed any other interventions by surgeon or anesthesiologist.

The anthropomorphic data included age, occupation; education level and physical condition based on ASA category were obtained for each patient. Blood Pressure (BP) and Heart Rate (HR) were measured at three times: before, during and after surgery (in recovery). Systolic BP lower than 100 mmHg was considered as hypotension, while, systolic or diastolic BP ≥140/90 mmHg was considered as hypertension. Also, HR higher than 100 and lower than 60 were considered as tachycardia and bradycardia, respectively. Moreover, respiratory depression was defined as O_2_ saturation ≤90. Measurement of patient satisfaction was performed in recovery by using Iowa Satisfaction Anesthesia Scale (ISAS) questionnaire with 11 options questions based on previous report. ISAS score more than 5.4 was considered as patient satisfaction (Fung, 2011).

Descriptive analysis was performed for reporting value in each parameter. Also, repeated measured ANOVA and mean rank comparison were performed for statistical analysis by using SPSS version 16.0. Independent sample t test for comparison between two treatment groups and Chi-square test for finding any correlation were used. P value under than 0.05 was considered as significant.

## 3. Results

Totally, 122 patients were included in this study at first, but seven patients from group ketamine+fentanyl and five patients from group propranolol were excluded due to lack of proper response in the questionnaire and therefore, 55 patients in each group were evaluated. These two groups were sex- and age-match (p>0.05). Frequency and percentage of patients in each group based on ASA category are presented in Table 1.

**Table 1:** The frequency and percentage of patients in both groups based on ASA category (n=110)

Groups	ASA category		

	I	II	III
Propranolol	10 (%18.2)	21 (%38.2)	24 (%43.6)
Ketamine + fentanyl	17 (%30.9)	25 (%45.5)	13 (%23.6)

Systolic, diastolic and arterial mean BP in each ASA category and each group in three different times of assessment are presented in Tables 2, 3 and 4, respectively.

**Table 2:** Mean and SD of systolic blood pressure (mmHg) in two groups based on different ASA category and in different time of assay

ASA category	Time of assay	Group	

		Propranolol	Ketamine +fentanyl
I	Before surgery	127.00±16.19	128.23±18.45
	During surgery	124.00±16.96	124.70±12.68
	After surgery	122.50±12.96	121.17±12.68
II	Before surgery	123.25±12.16	133.84±19.25
	During surgery	124.50±9.58	134.42±17.22
	After surgery	121.75±9.49	120.96±11.13
III	Before surgery	127.29±15.53	137.30±19.64
	During surgery	131.45±16.51	133.84±12.60
	After surgery	125.62±12.62	126.15±9.60

**Table 3:** Mean and SD of diastolic blood pressure (mmHg) in two groups based on different ASA category and in different time of assay

ASA category	Time of assay	Group	

		Propranolol	Ketamine +fentanyl
I	Before surgery	76.00±5.16	77.50±5.87
	During surgery	72.00±9.18	74.41±8.63
	After surgery	72.00±7.52	71.76±7.89
II	Before surgery	74.25±6.12	77.88±8.38
	During surgery	74.00±5.98	77.88±9.29
	After surgery	72.75±4.72	73.26±7.20
III	Before surgery	77.50±9.44	76.92±10.31
	During surgery	78.75±10.45	77.30±9.70
	After surgery	76.04±9.55	77.69±8.06

**Table 4:** Mean and SD of arterial mean blood pressure (mmHg) in two groups based on different ASA category and in different time of assay

ASA category	Time of assay	Group	

		Propranolol	Ketamine +fentanyl
I	Before surgery	93.00±8.19	94.11±9.46
	During surgery	89.33±10.31	91.17±8.91
	After surgery	88.83±7.97	88.23±8.50
II	Before surgery	90.58±7.32	96.53±11.04
	During surgery	90.83±6.17	96.73±10.35
	After surgery	89.08±5.14	89.16±7.38
III	Before surgery	94.09±11.03	97.05±12.65
	During surgery	96.31±11.37	96.15±9.01
	After surgery	92.56±10.04	93.84±7.43

**Figure 1:**
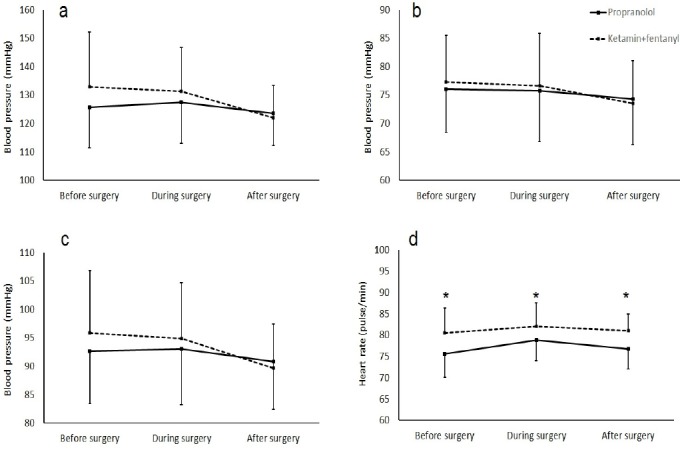
Comparison of mean and SD of systolic (a), diastolic (b), arterial mean (c) blood pressure and heart rate (d) in different groups at different time of assessment. Significant difference between two groups was indicated by asterisk.(P=0.001)

Comparison of mean and SD of systolic, diastolic and arterial mean BP and also HR in different groups at different time of assessment without considering ASA category are showed in Figure 1.

There were no significant differences between propranolol and ketamine+fentanyl groups in percentage of O_2_ saturation before (97.63±1.09 vs. 97.54±1.15 %, respectively, p=0.67) and during surgery (98.34±0.79 vs. 98.10±1.13 %, respectively, p=0.20). Comparison of frequency and percentage of incooperation, pain feeling and nausea during the surgery and also patient’s satisfaction after surgery between two groups are presented in Table 5. Comparison of ISAS score between propranolol and ketamine+fentanyl groups showed no significant difference (4.48±0.79 vs. 4.47±0.74 %, respectively, p=0.62).

**Table 5:** Frequency and percentage of incooperation, pain feeling and nausea during the surgery and patient’s satisfaction after surgery in two groups

Parameters	Groups	Frequency (percentage)	P value
Incooperation	Propranolol	4 (7.3)	0.72
	Ketamine + fentanyl	5 (9.1)	
Pain feeling	Propranolol	19 (34.5)	0.29
	Ketamine + fentanyl	13 (23.6)	
Nausea	Propranolol	5 (9.1)	<0.001
	Ketamine + fentanyl	23 (41.8)	
Patient’s satisfaction	Propranolol	49 (81.9)	0.29
	Ketamine + fentanyl	52 (94.5)	

In both groups, all surgical procedures were continued and finished without any problems. Also, there was no significant difference between propranolol and ketamine+fentanyl groups in duration of surgery (21.27±3.75 vs. 21.81±3.64 min, respectively, p=0.44).

## 4. Discussion

This study compared the BP and HR changes, duration of surgery, complication and patient’s satisfaction in response to using propranolol with routine ketamine+fentanyl as premedication for cataract surgery. Our results demonstrated that HR and probability of nausea in using ketamine+fentanyl were significantly higher than propranolol as premedication. Also, premedication with propranolol can induced reasonable and comparable satisfaction and painlessness against ketamine+fentanyl and be without any complications. Therefore, propranolol can be used instead of ketamine+fentanyl due to inexpensiveness and producing favorable results.

The number of reports about using of propranolol as premedication for cataract surgery is scarce and most use of propranolol in the field of eye diseases is related to treatment of hemangioma (Xue & Hildebrand, 2012; Missoi, 2011; Al-Dhaybi, 2011). However, a recent study was performed which discussed the pharmacokinetics and local safety profile of propranolol eye drops in animal model rabbits. They demonstrated that propranolol eye drops are able to ensure high retinal and low plasma concentrations of propranolol (Padrini, 2014). Also, the safety and efficacy of oral propranolol administration in preterm newborns affected by an early phase of retinopathy of prematurity (ROP) were evaluated and demonstrated that the administration of oral propranolol is effective in counteracting the progression of ROP but that safety is a concern (Filippi, 2013).

Anxiety before surgery can stimulate catecholamine secretion and induce hemodynamic inconstancy. Using of sedative drugs as premedication can overcome this anxiety and prepare patient for surgery. However, adjuvant intravenous anesthetic agents such as opioids, benzodiazepines, and other hypnotic sedative agents used to decrease pain and alleviate anxiety are associated with increases in medical events (Katz, 2001). The most important complications of these drugs include hypotension, bradycardia, respiratory and CNS depression, decline in arterial O2 saturation and also incooperation of patients (Ahmad, 2008). On the other hand, hypertension is the most common, and approximately half of the cataract patients have hypertension. In addition, patients with HR perform cataract surgery approximately twice more often than other patients. Also, BP tends to rise during cataract surgery (Çakmak, 2014). Propranolol as a beta adrenergic blocker can overcome most of hemodynamic features of anxiety and it has attracted the attention of physicians for using as premedication before surgery. Elman et al. reported that propranolol, 40 mg, administered 1 hour prior to surgery, significantly decreased anxiety in both surgeon and patients (Elman, 1998). Also, in another study, Gopal and his colleagues reported that propranolol (40 mg) given 2 hours prior to cataract surgery under local anesthesia reduces perioperative rise in HR and BP. Also, they reported that relative to controls, cases showed lower mean pulse rate and mean systolic BP at all-time intervals. They concluded propranolol effectively reduces anxiety before and during the surgery (Gopal, 2005). Our results are in agreement with all other previous reports and demonstrated the beneficial effects of propranolol as premedication for cataract surgery.

## 5. Conclusion

In conclusion our results demonstrated that propranolol can be used as premedication for cataract surgery without any complication and also provide similar and sometimes more efficacy than routine ketamine plus fentanyl premedication. However, further studies in different age group and also different eye disease are needed to confirm the findings of this study.
